# Isolation and pathogenicity of *Xylella fastidiosa* associated to the olive quick decline syndrome in southern Italy

**DOI:** 10.1038/s41598-017-17957-z

**Published:** 2017-12-18

**Authors:** M. Saponari, D. Boscia, G. Altamura, G. Loconsole, S. Zicca, G. D’Attoma, M. Morelli, F. Palmisano, A. Saponari, D. Tavano, V. N. Savino, C. Dongiovanni, G. P. Martelli

**Affiliations:** 1 Consiglio Nazionale delle Ricerche-Istituto per la Protezione Sostenibile delle Piante (CNR-IPSP), Sede Secondaria di Bari, 70126 Bari, Italy; 2 Università degli Studi di Bari Aldo Moro, Dipartimento di Scienze della Pianta, del Suolo e degli Alimenti (DiSSPA), 70126 Bari, Italy; 3 Centro di Ricerca, Formazione e Sperimentazione in Agricoltura (CRSFA) “Basile Caramia”, 70010 Locorotondo (Bari), Italy

## Abstract

In autumn 2013, the presence of *Xylella fastidiosa*, a xylem-limited Gram-negative bacterium, was detected in olive stands of an area of the Ionian coast of the Salento peninsula (Apulia, southern Italy), that were severely affected by a disease denoted olive quick decline syndrome (OQDS). Studies were carried out for determining the involvement of this bacterium in the genesis of OQDS and of the leaf scorching shown by a number of naturally infected plants other than olive. Isolation in axenic culture was attempted and assays were carried out for determining its pathogenicity to olive, oleander and myrtle-leaf milkwort. The bacterium was readily detected by quantitative polymerase chain reaction (qPCR) in all diseased olive trees sampled in different and geographically separated infection foci, and culturing of 51 isolates, each from a distinct OQDS focus, was accomplished. Needle-inoculation experiments under different environmental conditions proved that the Salentinian isolate De Donno belonging to the subspecies *pauca* is able to multiply and systemically invade artificially inoculated hosts, reproducing symptoms observed in the field. Bacterial colonization occurred in prick-inoculated olives of all tested cultivars. However, the severity of and timing of symptoms appearance differed with the cultivar, confirming their differential reaction.

## Introduction

Olive quick decline syndrome (OQDS) is a disease emerged at the end of the first decade of the XX century in a restricted area of the Ionian coast of Salento (Apulia, southeastern Italy); but the disease incidence increased rapidly through the heavily olive-grown countryside of the peninsula^1^. Search for the causal agent(s) of this new disorder disclosed the consistent presence in diseased olive trees, as well as in other hosts exhibiting leaf scorch symptoms, of *Xylella fastidiosa* Wells, Raju *et al*. 1986, a xylem-limited Gram-negative gammaproteobacterium^2^.

*X*. *fastidiosa* has a long phytosanitary history in the Americas, where it is known as the agent of economically important diseases such as Pierce’s disease of grapevine, citrus variegated chlorosis, and a number of disorders of perennial crops and landscape plants^3^. In Apulia, however, olive trees appear to be the host of major economic relevance, reacting to infection with scattered desiccations of twigs and small branches that show first on the upper part of the crown, then extend to the rest of the canopy, which acquires a blighted aspect. OQDS attacks lead to the death of the trees within a few years from the onset of the symptoms. The highly susceptible local cultivars ‘Cellina di Nardò’ and ‘Ogliarola salentina’ are the most severely affected. These symptomatic trees are subjected to heavy pruning to stimulate new growth, but the new vegetation soon withers and desiccates^1^.

Before 2013, the only study of *X*. *fastidiosa* infecting olives, was from California (USA), where Krugner *et al*.^4^ detected *X*. *fastidiosa* subsp. *multiplex* in trees with leaf scorch and branch dieback. Isolates recovered from some of these symptomatic trees were successful inoculated and vector-transmitted to olive plants of different cultivars, but under experimental conditions infections did not cause symptoms resembling those observed in the field. It was only after the discovery of *X*. *fastidiosa* in southern Italian olives, that more in-depth investigations were carried out on this crop in Argentina and Brazil, where, symptomatic plants were also found to host *X*. *fastidiosa* subsp. *pauca* ^5,6^, i.e. the same subspecies to which the Italian olive-infecting strain belongs to^7,8^.

A recent study by Strona *et al*.^9^ underlined that the wide distribution of olive orchards in Apulia and the abundance of a bacterial vector (*Philaenus spumarius* L.) populations^10^ are the factors that contribute the most to the entrenchment of *X*. *fastidiosa* in the territory and to the emergence of the associated disease. This alarming scenario is further aggravated by the favourable conditions for bacterial growth and host colonization occurring in the coastal areas of the Mediterranean, which are characterized by a temperate climate with mild winters^11,12^.

The well-documented detrimental impact of *X*. *fastidiosa* on a number of major crops, and its recent appearance in southern Italy^13^, Corsica and mainland France^14^, and Balearic Islands of Spain^15^, represent a serious threat to the Mediterranean countries, whose major agricultural industries, i.e. olive, grapevine, stone fruits and citrus are endangered, as well as a number of landscape and forest species. This phytosanitary emergency has thus called for the implementation of research programmes to secure knowledge on the interactions of this exotic bacterium with the newly colonized environment and the possible hosts. In response to this knowledge gap and the devastation caused by OQDS, a campaign was initiated to isolate and culture the bacterium from affected plants and use mechanical inoculation to determine its pathogenicity, extending preliminary studies^16^. To determine *X*. *fastidiosa*-OQDS relationships bacterial isolations in axenic culture were done. Cultured bacteria were then used for mechanical inoculation of olive (*Olea europaea*), oleander (*Nerium oleander*) and myrtle-leaf milkwort (*Polygala myrtifolia*) plantlets to assess their ability to colonize these hosts and reproduce the symptoms observed in the field.

## Results

### Detection and culture of *X*. *fastidiosa* from symptomatic olive trees

*X*. *fastidiosa* was detected by qPCR in all fifty-eight symptomatic trees sampled in the oldest (2013) and the more recent (2016) OQDS outbreaks. Quantitative PCR reactions yielded quantitative cycles (Cq) in the range of 21–26, which corresponded to an average population of 4.5–6 (log cells/gr of tissue when interpolated on the linear standard curve generated using 10-fold serial dilutions of a bacterial suspension with known concentration). Fluorescence signal in the negative and non-template controls remained below the background, with no Cq values.

**Figure 1:**
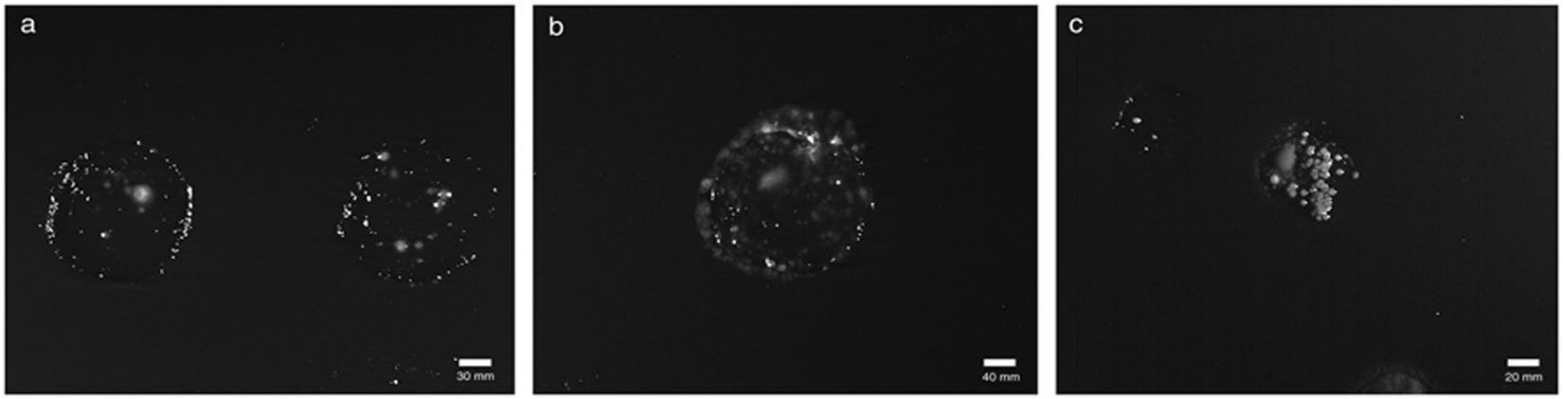
*Xylella fastidiosa* colonies on BCYE agar medium growing in different stem-prints obtained after imprinting the fresh cut surface of the olive cuttings on the medium. (**a**) Shows a low number of colonies per spot; (**b**) and (**c**) show the high number of colonies growing mostly together.

Actively growing *X*. *fastidiosa* colonies were successfully obtained from 51 of the 58 olive samples, collected in distinct OQDS foci (Table 1). Colonies grew relatively slowly, requiring 7 to 15 days to become visible (Fig. 1) and developed in several spotted imprints of each sample, an indication of the wide distribution of the bacterium in symptomatic trees. The highest number of spots per tree with actively growing colonies (i.e. 10–30 spots/tree) was obtained in May-June, whereas isolations made in August and January yielded colonies in fewer spots (i.e. 3–4 spots/tree). All cultured isolates were confirmed as *X*. *fastidiosa* by qPCR and were triple cloned prior to being stored in glycerol at −80 °C.

**Table 1:** List of the olive groves showing symptoms of Olive quick decline syndrome (OQDS) and representing the different foci used to collect the olive samples for the identification of *Xylella fastidiosa* by quantitative PCR (qPCR) (Harper *et al*.^36^) and isolation in axenic culture. In each grove, three symptomatic trees were selected and sampled.

Foci OQDS	Code of the samples	Municipality (Province)	Date of sampling	Results of the qPCR for *X*. *fastidiosa* ^*a*^	Cultured isolate
APL-1	WPT 1129	Minervino di Lecce (Lecce)	May, 2016	Positive	YES
APL-2	WPT 1130	Uggiano la Chiesa (Lecce)	May, 2016	Positive	YES
APL-3	WPT 1133	Cursi (Lecce)	May, 2016	Positive	YES
APL-4	WPT 1134	Supersano (Lecce)	May, 2016	Positive	YES
APL-5	WPT 1135	Maglie (Lecce)	May, 2016	Positive	YES
APL-6	WPT 1140	Muro Leccese (Lecce)	June, 2016	Positive	YES
APL-7	WPT 1141	Palmariggi (Lecce)	June, 2016	Positive	YES
APL-8	WPT 1144	Spongano (Lecce)	June, 2016	Positive	YES
APL-9	WPT 1145	Andrano (Lecce)	June, 2016	Positive	YES
APL-10	WPT 1148	Tricase (Lecce)	June, 2016	Positive	YES
APL-11	AS	Cutrofiano (Lecce)	June, 2016	Positive	YES
APL-12	AV	Avetrana (Taranto)	June, 2016	Positive	YES
APL-13	CS	Campi Salentina (Lecce)	June, 2016	Positive	YES
APL-14	CIST	Alliste (Lecce)	May, 2016	Positive	YES
APL-15	CUT	Cutrofiano (Lecce)	June, 2016	Positive	YES
APL-16	FP	Presicce (Lecce)	June, 2016	Positive	YES
APL-17	GC	Gagliano del capo (Lecce)	June, 2016	Positive	YES
APL-18	GD	Gagliano del capo (Lecce)	June, 2016	Positive	YES
APL-19	Gigante	Alliste (Lecce)	May, 2016	Positive	YES
APL-20	Giug	Giuggianello (Lecce)	May, 2016	Positive	YES
APL-21	La Castellana	Matino (Lecce)	June, 2016	Positive	YES
APL-22	San CAS	San Cassiano (Lecce)	May, 2016	Positive	YES
APL-23	TK	Nociglia (Lecce)	May, 2016	Positive	YES
APL-24	SP1	Morciano di leuca (Lecce)	June, 2016	Positive	YES
APL-25	SP3	Salve (Lecce)	June, 2016	Positive	YES
APL-26	SP4	Presicce (Lecce)	June, 2016	Positive	YES
APL-27	SP7	Specchia (Lecce)	June, 2016	Positive	YES
APL-28	Dedonno (CFBP 8402)	Gallipoli (Lecce)	June, 2014	Positive	YES
APL29	SZ	Squinzano (Lecce)	May, 2016	Positive	YES
APL-30	TR	Alliste (Lecce)	May, 2016	Positive	YES
APL-31	UG	Ugento (Lecce)	May, 2016	Positive	YES
APL-32	ORIA	Oria (Brindisi)	June, 2016	Positive	YES
APL-33	VEG	Veglie (Lecce)	June, 2016	Positive	YES
APL-34	CU	Cutrofiano (Lecce)	June, 2016	Positive	YES
APL-35	FO	Taviano (Lecce)	June, 2016	Positive	YES
APL-36	VN	Gallipoli (Lecce)	June, 2016	Positive	YES
APL-37	ST	Sternatia (Lecce)	June, 2016	Positive	YES
APL-38	GA	Gagliano del capo (Lecce)	June, 2016	Positive	YES
APL-39	MELC A	Ugento (Lecce)	August, 2014	Positive	YES
APL-40	COP	Copertino (Lecce)	August, 2014	Positive	YES
APL-41	CUR	Cursi (Lecce)	August, 2014	Positive	YES
APL-42	SC	Presicce (Lecce)	August, 2014	Positive	YES
APL-43	LI SAULI	Gallipoli (Lecce)	June, 2016	Positive	YES
APL-44	WPT 1137	Salve (Lecce)	May, 2016	Positive	YES
APL-45	WPT 1139	Specchia (Lecce)	May, 2016	Positive	NO
APL-46	WPT 1142	Otranto (Lecce)	June, 2016	Positive	YES
APL-47	WPT 1143	San Cassiano (Lecce)	June, 2016	Positive	YES
APL-48	WPT 1146	Specchia (Lecce)	June, 2016	Positive	NO
APL-49	WPT 1147	Alessano (Lecce)	June, 2016	Positive	NO
APL-50	SP2	Salve (Lecce)	June, 2016	Positive	NO
APL-51	SP5	Miggiano (Lecce)	June, 2016	Positive	NO
APL-52	SP6	Montesano Salentino (Lecce)	June, 2016	Positive	NO
APL-53	SP8	Specchia (Lecce)	June, 2016	Positive	NO
APL-54	FP	Presicce (Lecce)	June, 2016	Positive	YES
APL-55	RAC	Racale (Lecce)	June, 2016	Positive	YES
APL-56	TR	Trepuzzi (Lecce)	June, 2016	Positive	YES
APL-57	SQ1	San Vito dei Normanni (Brindisi)	January, 2017	Positive	YES
APL-58	SQ2	Carovigno (Brindisi)	January, 2017	Positive	YES

^a^Samples were assessed as “Positive” when qPCR reactions produced quantitative cycle (Cq) > 0 and < 32; “Doubtful” with Cq > 32; “Negative” when no fluorescence was detected in the reaction, Cq = 0.

### Multiplication and movement of *X*. *fastidiosa* in olive trees

*X*. *fastidiosa* was successfully prick-inoculated in olive stems. Bacterial multiplication and movement occurred in the inoculated plants of all cultivars, regardless of whether they were grafted (‘Cellina di Nardò’) or self-rooted (’Leccino’, ‘Coratina’ and ‘Frantoio’).

Time-course qPCR assays, however, disclosed a differential rate of bacterial colonization in inoculated plants grown in a greenhouse under temperature controlled conditions (Experiment A) or in a net tunnel (Experiment B), thus exposed to seasonal temperature fluctuations. In all experiments, consistent negative results were obtained when leaf petioles were tested from mock-inoculated plants, all of which remained symptomless (Tables 2 and 3).

**Table 2:** Detection of *Xylella fastidiosa* from plant tissues collected at various distances from the inoculation points after needle-inoculation. Sampling was performed at 1, 3, 6, 9, 12 and 24 months post inoculation (mpi). Experiment A and B were performed in a quarantine-equipped glasshouse and in a screen net tunnel, respectively.

Cultivar	Leaf and stem tissues														Roots	
	1 mpi		3 mpi					6 mpi		9 mpi			12 mpi	24 mpi^c^	12 mpi^d^	24 mpi^e^
	IP^a^	1st^b^	IP	1st	2nd	3rd	4th	5th	6th	6th	7th	8th	AP^f^	AP		
**Experiment (A)**																
CELLINA DI NARDO’	7/10	2/10	7/10	4/10	4/10	2/10	0/10	9/10	0/10	9/10	0/10	nt^g^	9/10	0/1	3/3	9/10
CORATINA	4/10	0/10	4/10	0/10	nt	nt	nt	2/10	0/10	2/10	0/10	nt	4/10	1/6	0/3	0/10
FRANTOIO	5/10	0/10	5/10	2/10	0/10	nt	ni	3/10	0/10	3/10	1/10	0/10	7/10	0/3	1/3	1/10
LECCINO	5/10	0/10	5/10	5/10	0/10	nt	nt	3/10	0/10	3/10	1/10	0/10	8/10	0/2	1/3	6/10
Mock inoculated controls (3 for each cultivar)	0/12	0/12	0/12	0/12	nt	nt	0/12	0/12	nt	nt	nt	0/12	0/12	0/12	0/3	0/10
**Cultivar**	**Leaf and stem tissues**														**Roots**	
	**1 mpi**		**3 mpi**			**6 mpi**		**9 mpi**			**12 mpi**		**24 mpi** ^**c**^		**12 mpi** ^**d**^	**24 mpi** ^**e**^
	**IP** ^**a**^	**1st** ^**b**^	**1st**	**2nd**	**3rd**	**2nd**	**3rd**	**4th**	**5th**	**6th**	**6th**	**AP** ^**f**^	**AP**			
**Experiment** (**B**)																
CELLINA DI NARDO’	7/10	4/10	4/10	1/10	0/10	3/10	3/10	3/10	3/10	0/10	3/10	0/10	4/7		0	3/10
CORATINA	6/10	0/10	0/10	0/10	nt	1/10	0/10	0/10	nt	nt	nt	0/10	0/7		0	0/10
FRANTOIO	5/10	0/10	0/10	0/10	nt	0/10	0/10	0/10	nt	nt	nt	0/10	0/7		0	0/10
LECCINO	6/10	0/10	1/10	0/10	nt	0/10	0/10	0/10	nt	nt	nt	0/10	1/7		0	0/10
Mock-inoculated controls (3 for each cultivar)	0/12	0/12	0/12	0/12	nt	nt	0/12	0/12	nt	nt	nt	0/12	0/7		0	0/10

^a^IP indicates inoculation point.

^b^1^st^–8^th^ = indicates the node above the IP.

^c^The figure indicates the number of positive plants when re-tested at 24 mpi among those testing negative at 12 mpi.

^d^Number of positive samples out of three subjected to the diagnostic test.

^e^Cumulative number of positive samples at 24 mpi.

^f^AP = apical portion of the inoculated shoots.

^g^Nt = not tested.

**Table 3:** Symptomatic shoots recovered on inoculated plants of ‘Cellina di Nardò’ (Experiment C). Incidence of shoots showing symptoms.

ID inoculated plant	Quantitative PCR results (7 months post inoculation -mpi)		% of shoots showing desiccation	
	Inoculated shoots^b^	Non-inoculated shoots^c^	12 mpi	24 mpi
X1	Positive	Positive	96.15	100.00
X2	Positive	Positive	80.77	100.00
X3	Positive	Positive	71.88	100.00
X4	Positive	Positive	76.92	100.00
X5	Positive	Positive	75.00	100.00
X6	Positive	Positive	55.26	67.39
X7^d^	Positive	Positive	77.27	100.00
X8	Positive	Positive	44.00	100.00
X9	Positive	Negative	13.33	20.75
X10^d^	Positive	Negative	27.91	28.85
X11	Positive	Positive	48.94	61.90
X12	Positive	Positive	58.62	73.53
X13	Positive	Positive	64.71	71.43
X14	Positive	Positive	40.63	42.50
^e^XHC1-XHC10	Negative (10 plants)	Negative (10 plants)	0	0

^a^Percentage referred to the number of shoots displaying symptoms of desiccation on the total number of shoots (approximately 25–30 per plant).

^b^Samples consisted of leaves taken from the shoots harbouring the inoculation points; the leaves were collected 25–30 cm above the inoculation points.

^c^Three shoots were randomly selected to determine if the bacterium spread from the inoculated shoots to the rest of the plants.

^d^See Fig. 5.

^e^Ten mock-inoculated plants.

#### Experiments A and B

One month post-inoculation (mpi), the percentage of qPCR-positive plants at the inoculation point (IP) ranged between 40 (4/10) and 70% (7/10) (Table 2, Experiment A and B) with the highest detection rate in ‘Cellina di Nardò’. At the same time, qPCR testing of the leaf from the node 2–3 cm above the IPs showed very limited bacterial movement, with only a few samples of ‘Cellina di Nardò’ testing positive (Table 2, Experiment A and B).

qPCR assays at different times post inoculation showed that greenhouse-grown plants of ‘Cellina di Nardò’ were more rapidly colonized by the bacterium than the other cultivars. In fact, in Experiment A, at 9 mpi *X*. *fastidiosa* was detected up to the 6^th^ internode above the IP (*ca*. 18 cm) in 9 of 10 inoculated plants, whereas the number of plants in which the bacterium had reached the same distance from the IP was much lower with other cultivars, i.e. two plants of ‘Coratina’ and three plants of ‘Leccino’ and ‘Frantoio’. In the remaining plants of these cultivars the bacterium remained confined between the 1^st^ and the 5^th^ internode (*ca*. 5–12 cm above the point of inoculation). These differences proved to be statistically significant (Supplementary Table S1). At 12 mpi *X*. *fastidiosa* was successfully detected in the leaves of the apical portion of 9, 8, 7 and 4 inoculated plants of ‘Cellina di Nardò’, ‘Leccino’, ‘Frantoio’ and ‘Coratina’, respectively. The number of infected plants for each cultivar did not vary at 24 mpi except for ‘Coratina’, which increased to 5 infected plants.

As shown in Table 2, when the roots of three systemically infected plants of ‘Cellina di Nardò’ were tested for the presence of *X*. *fastidiosa* at 12 mpi the bacterium was detected in the roots of all of them, contrary to what was observed for ‘Coratina’, ‘Leccino’ and ‘Frantoio’. The overall results at 24 mpi were in line with those recorded at 12 mpi, i.e. roots from 9 of 10 ‘Cellina di Nardò’ and 6 of 10 ‘Leccino’ were positive, while the roots of ‘Coratina’ remained *Xylella*-free and a very limited infection (1/10) was detected in ‘Frantoio’.

Bacterial re-isolation was successfully accomplished from infected plants of all four cultivars. Colonies were recovered from all sections of the stems, with the highest number of colonies per spot recovered from the distal portions above the inoculum points. These different portions were also assayed by qPCR, showing that *X. fastidiosa* was consistently detected from both stem and leaves sampled. However, bacterial population estimates, based on the interpolation on the standard curve generated using 10-fold serial dilutions, varied with the cultivar (Table 4, Supplementary Table S2) and, for ‘Cellina di Nardo’, between plants maintained under controlled or uncontrolled temperature conditions. Infected plants of ‘Cellina di Nardò’ hosted the highest bacterial populations, ranging from a minimum of 2.79E + 06 in leaf petioles to a maximum of 1.15E + 07 CFU/ml in the stem. Average ranging from 6.38E + 04 to 5.65E + 05 were obtained for the infected plants of the remaining cultivars.

**Table 4:** Average of the bacterial concentration expressed as CFU/g of plant tissue determined by quantitative PCR in the portions of the three plants subjected to destructive sampling. Plants were subdivided into 4 portions (from the bottom to the top), the stem and the leaves of each portion were tested separately. The average of the bacterial concentration for each type of tissue was calculated and subjected to statistical analysis. The standard error of the mean (SEM) is used to describe the variability within the sample.

OLIVE CULTIVARS	Portion of the plant								Average^1^	
	1st (Bottom)		2nd		3rd		4th (Apical)			
	Stem	Leaf petioles	Stem	Leaf petioles	Stem	Leaf petioles	Stem	Leaf petioles	Stem	Leaf petioles
Cellina di Nardò	1.15E + 07	2.79E + 06	2.99E + 06	2.85E + 06	1.02E + 07	2.79E + 06	2.32E + 06	5.55E + 06	6.77E + 06 ± 2.39E + 06 a	3.50E + 06 ± 6.84E + 05 a
Leccino	6.69E + 04	8.10E + 03	5.42E + 04	1.35E + 05	9.06E + 04	2.78E + 05	4.35E + 04	6.98E + 05	6.38E + 04 ± 1.01E + 04 b	2.54E + 05 ± 1.41E + 05 b
Frantoio	6.35E + 05	1.13E + 05	5.64E + 05	2.89E + 04	5.01E + 05	3.72E + 05	1.43E + 05	1.76E + 05	4.71E + 05 ± 1.18E + 05 b	1.67E + 05 ± 7.31E + 04 b
Coratina	3.45E + 04	7.45E + 04	6.08E + 05	5.41E + 04	8.57E + 05	6.88E + 04	7.59E + 05	1.06E + 05	5.65E + 05 ± 1.84E + 05 b	7.59E + 04 ± 1.09E + 04 b

^1^One-way ANOVA comparing the bacterial concentration among the four cultivars. Different letters represent significant differences between means at p < 0.05, according to Tukey’s HSD post-hoc test.

qPCR assays on the samples collected in the time-course experiment under uncontrolled temperature conditions (Experiment B, plants grown under a net tunnel) yielded a significant lower number of positive reactions (Table 2), than in the plants maintained under controlled temperature conditions, likely because bacterial colonization of potted plants was substantially affected by the variable climatic conditions at which these plants were exposed. However, ‘Cellina di Nardò still proved to be the most susceptible to *X*. *fastidiosa*, with three and four plants systemically infected at 12 and 24 mpi, respectively, including roots of three of the four infected plants. At 24 mpi only one plant of ‘Leccino’ was systemically infected, and none of those of ‘Frantoio’ and ‘Coratina’. The bacterium was not detected in the roots of any of these three cultivars.

Bacterial population estimates in the four infected plants of ‘Cellina di Nardò’ grown under the net tunnel, yielded an average value of 2.98E + 05 ± 1.96 + 05, a figure lower than those estimated for greenhouse-grown plants of the same cultivar (Supplementary Table S3).

#### Experiment C

qPCR reactions 1 mpi were positive at the IPs for all the inoculated plants as well as at 7 mpi, when the bacterium was detected in all samples collected 10–15 cm above the IPs. At the same time, *X*. *fastidiosa* was successfully detected in non-inoculated shoots 12 out of 14 inoculated plants (Table 3).

### Symptom progression in inoculated olive plants

**Figure 2:**
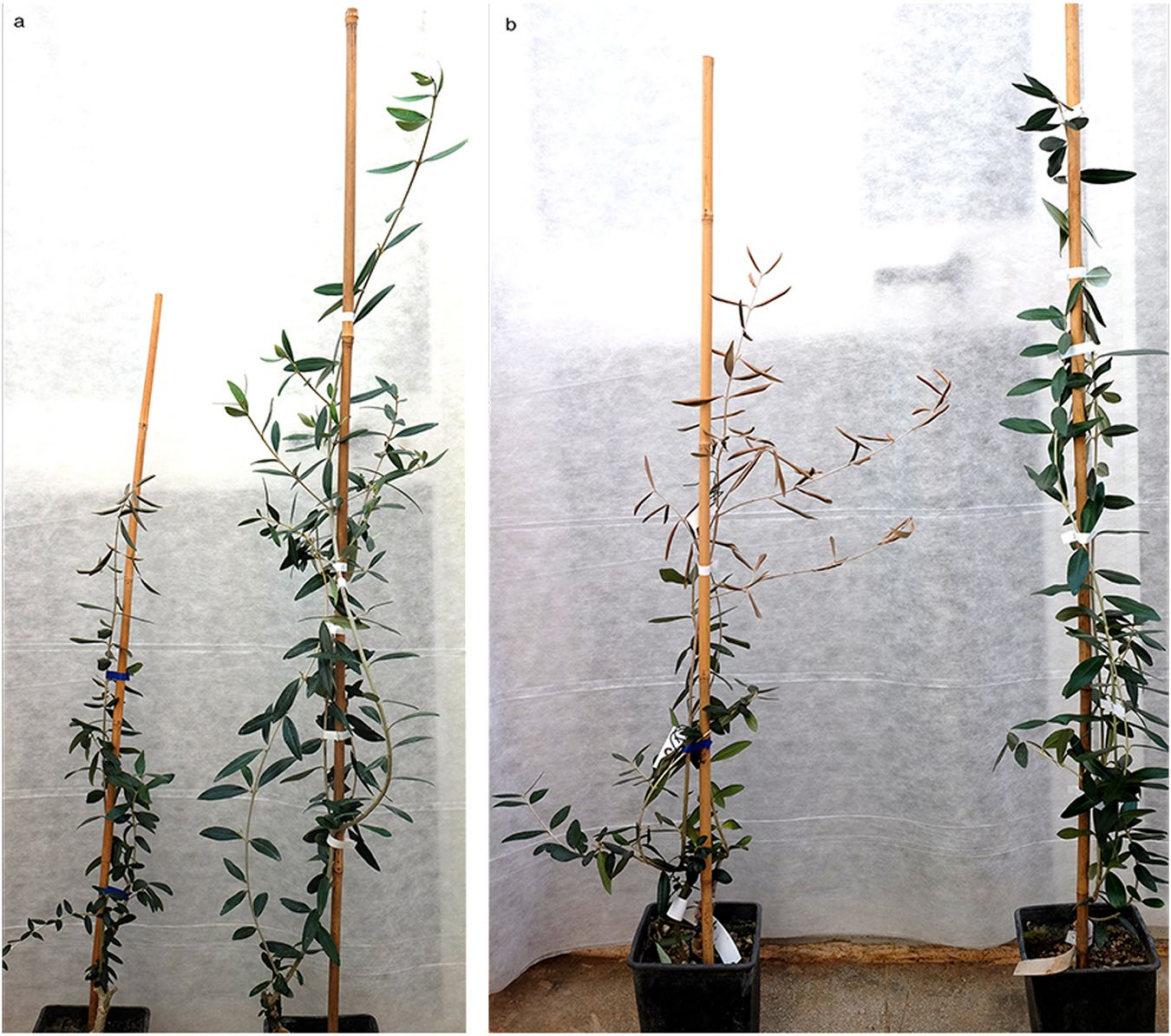
Inoculated plant of ‘Cellina di Nardò’. (**a**) Plants 12 months post inoculation, on the left panel a systemically infected plant showing reduced growth, wilting and desiccation starting from the apical portion of the shoot. Mock-inoculated control on the right end side. (**b**) Severe symptoms of desiccation progressing rapidly in an infected plant 14 months post inoculation. The mock-inoculated plant (right end site) is symptomless.

Under greenhouse conditions, the evolution of symptoms was similar to that observed in the field: leaves became first chlorotic, then withered, turned brown and desiccated. Symptoms consistently started from the apical portion of the inoculated shoots and progressed toward their base (Fig. 2a).

#### Experiment A

**Figure 3:**
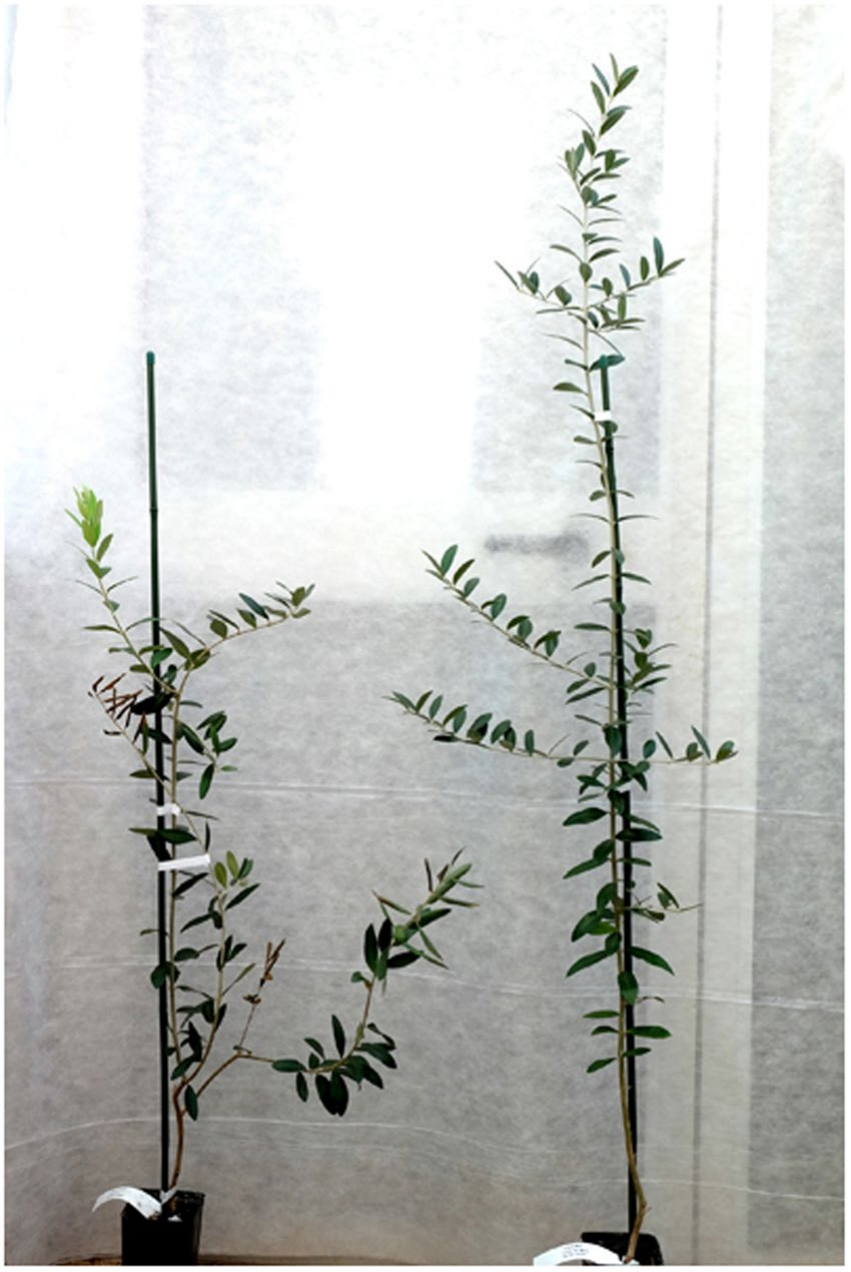
Stunting and limited desiccation of a *X*. *fastidiosa*-inoculated ‘Leccino’ plant. Symptomless mock-inoculated ‘Leccino’ on the right end side.

Periodical visual inspections did not show any apparent reactions up to 12 mpi, when initial symptoms of wilting, followed by desiccation and dieback were observed in two of the inoculated plants of ‘Cellina di Nardò’. During the following couple of months (13–14 mpi) these symptoms rapidly progressed and appeared also on the remaining plants of the same cultivar, except for one in which the bacterium remained confined at the inoculation point. The desiccated part of inoculated and non-inoculated shoots ranged from 30 to 100% of the entire shoot length (Table 5, Fig. 2b). At about the same time, desiccation began to appear on two and three plants of ‘Frantoio’ and ‘Leccino’, respectively (Table 5, Fig. 3). None of the plants of ‘Coratina’ showed symptoms up to 14 mpi.

**Table 5:** Presence and intensity of the symptoms recorded in the plants systemically infected by *Xylella fastidiosa*. The data refer to the number of plants (out of a total of ten) that showed symptoms within the first 14 months post inoculation (mpi) and at 24 mpi. Three plants were destructively sampled at 14 mpi thus the total plants at 24 mpi do not correspond to the original inoculated plants. Reduction in growth was determined by measuring the plant size (expressed as height) of the symptomatic plants and mock-inoculated controls. F-test in One-Way ANOVA was used and outputs for ANOVA and Tukey’s HSD post-hoc tests are reported in the Supplementary Table S4.

CULTIVAR	PLANTS SHOWING DESICCATION	% OF THE CANOPY AFFECTED BY DESICCATION^1^	PLANTS SHOWING DESICCATION^2^	% OF THE CANOPY AFFECTED BY DESICCATION^3^	REDUCTION IN PLANT SIZE^4^ (%)
	14mpi		24mpi		
CELLINA DI NARDO’	7/10	44.8 ± 14.3 a	6/7	85.7 ± 14.3 a	36.5
CORATINA	0/10	0.0 ± 0.0 b	2/7	7.4 ± 5.1 b	17.9
FRANTOIO	2/10	4.0 ± 2.7 b	5/7	23.3 ± 7.2 b	24.9
LECCINO	3/10	1.9 ± 0.9 b	6/7	42.0 ± 8.6 b	7.5

^1,3^Average values. These values were calculated by measuring the length (cm) of the symptomatic portion of each shoot on the total length (cm) of the shoots present on each plant. The standard error of the mean (SEM) is used to describe the variability within the sample.

^2^Number of plants showing symptoms at 24 mpi over the total number of plants under observation after the destructive sampling.

^1,3^Different letters represent significant differences between mean values at p < 0.05, according to one-way ANOVA comparison, followed by Tukey’s HSD post-hoc test.

^4^Values are expressed in percentage and indicate the height difference between mock-inoculated and symptomatic plants.

**Figure 4:**
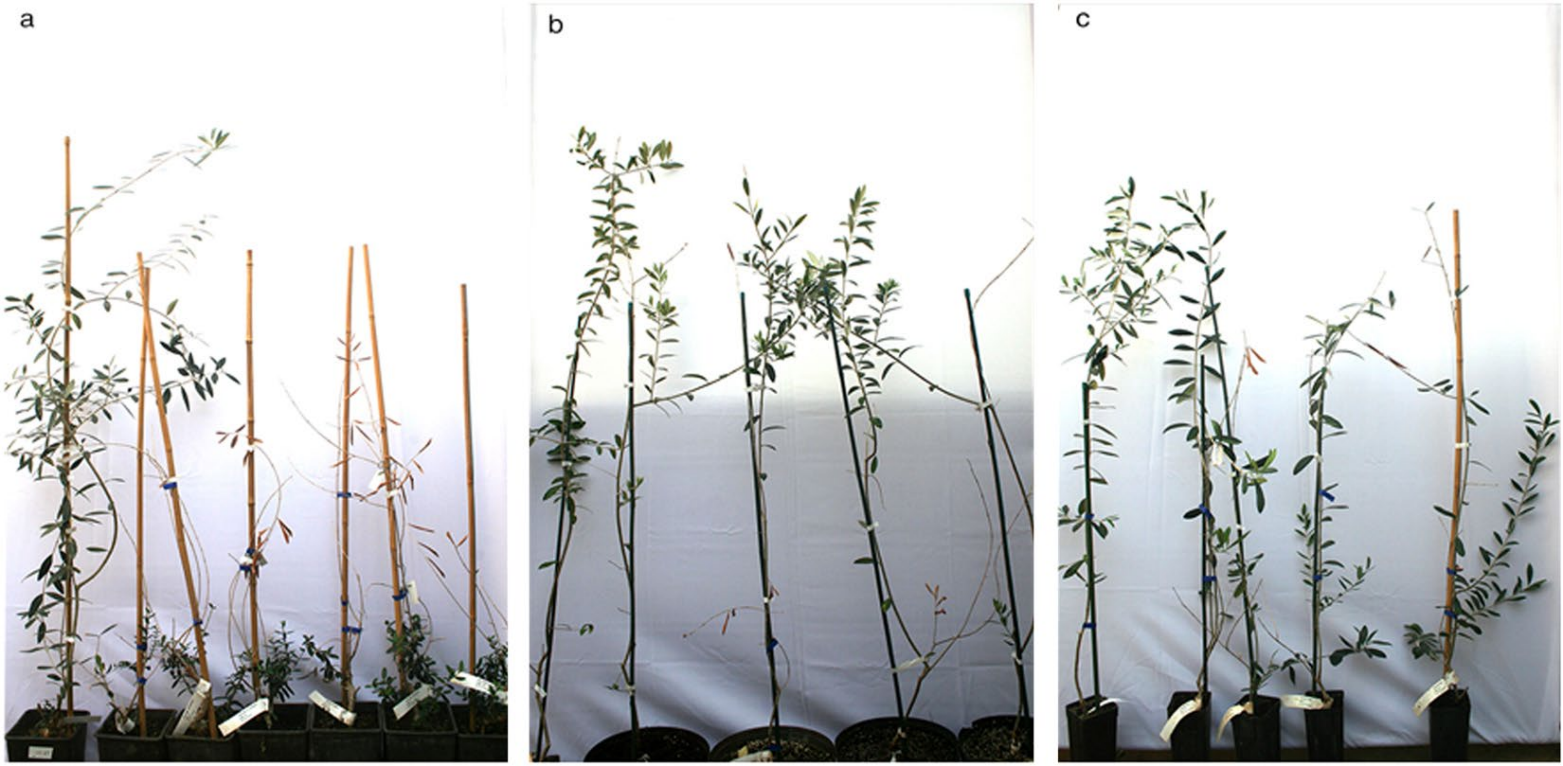
Olive plants 24 months post inoculation. In all panels the first plant on the left is the mock-inoculated symptomless control. (**a**) All grafted plants of ‘Cellina di Nardò’ are desiccated except for the suckers pushed by the rootstocks which are symptomless, as it often occurs in the field. Plants of ‘Leccino’ (**b**) and ‘Frantoio’ (**c**) much less affected that those of the highly susceptible ‘Cellina di Nardò’.

At 24 mpi, all plants of ‘Cellina di Nardò’ displayed completely desiccated shoots. Some symptomless sprouts were pushed by the rootstocks (Table 5, Fig. 4a), i.e. a condition comparable to that observed in naturally infected field plants. Symptom appearance was delayed in ‘Leccino’ (Fig. 4b), and, at the same date, symptoms had not progressed much in ‘Frantoio’ (Table 5, Fig. 4c).

In general, symptoms began to show 3–5 months after the systemic invasion of the artificially inoculated plants, in agreement with field observations that suggested the existence of a time lag between the timing of infection and appearance of symptoms.

#### Experiment B

Up to 24 mpi no distinct symptoms could be observed in the inoculated plants of this experiment, a condition likely linked to the poor colonization and the low bacterial population revealed by qPCR assays.

#### Experiment C

**Figure 5:**
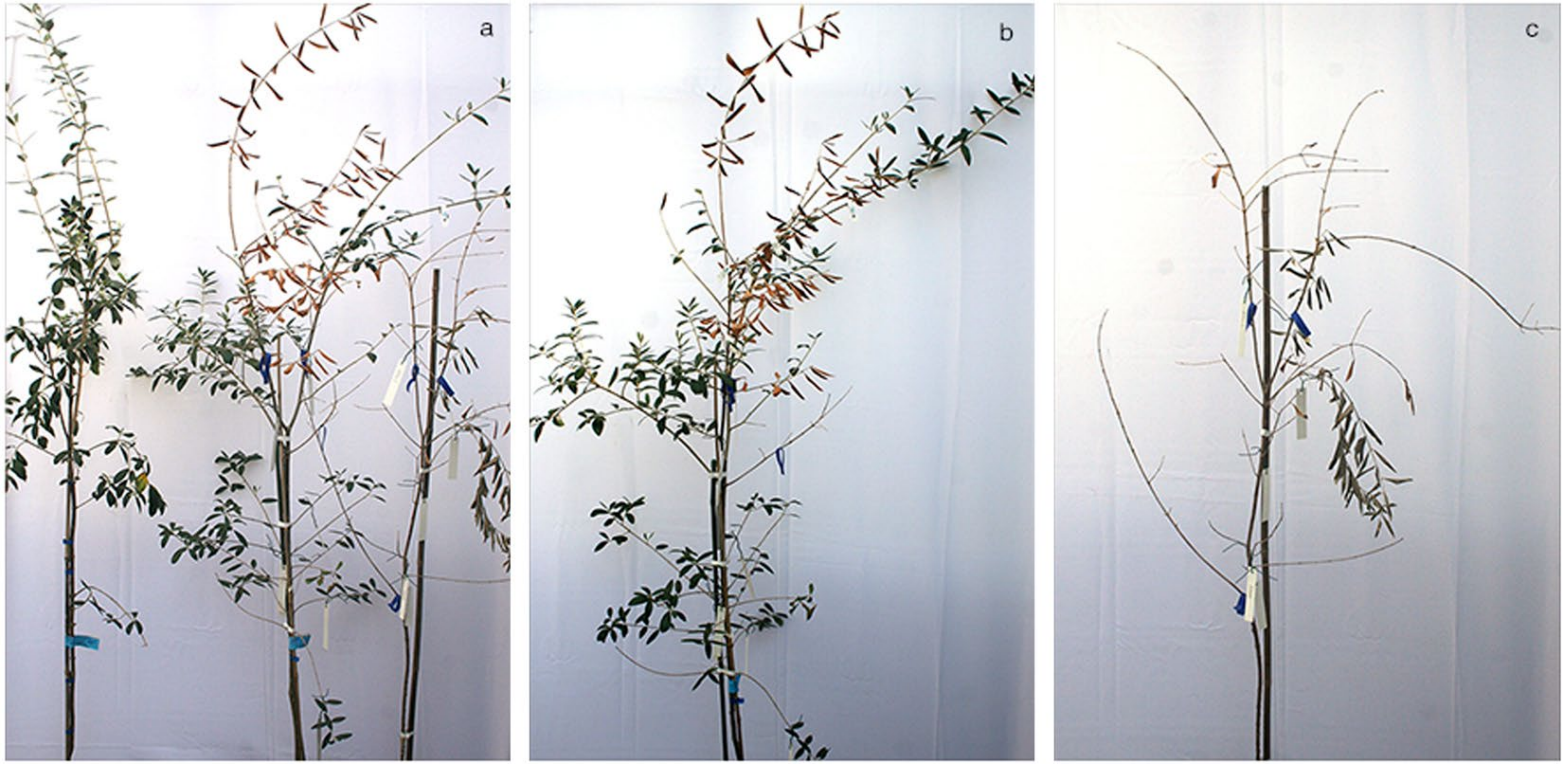
Inoculated plants of ‘Cellina di Nardò’ 24 months post inoculation (Experiment C). (**a**) Severely damaged plants on the right of the mock-inoculated control; (**b**) and (**c**) infected plants × 7 and × 10 (see Table 3).

Symptoms similar to those observed in ‘Cellina di Nardò’ in Experiment A, developed 8–10 mpi in all the inoculated plants, starting from the top of the canopy and progressing rapidly. Thus, at 24 mpi the totality of the shoots of most plants displayed dieback and desiccation (Table 3) (Fig. 5). None of such symptoms was seen in the mock-inoculated controls.

### Symptoms, multiplication and movement of *X*. *fastidiosa* in oleander and myrtle-leaf milkwort

Mechanical inoculation of *X*. *fastidiosa* in oleander and myrtle-leaf milkwort grown under greenhouse conditions (Experiment A) resulted in effective systemic invasion and symptom development in both hosts. By converse, mechanical infections of plants grown under the net tunnel did not succeed, and no symptoms developed (Experiment B).

**Figure 6:**
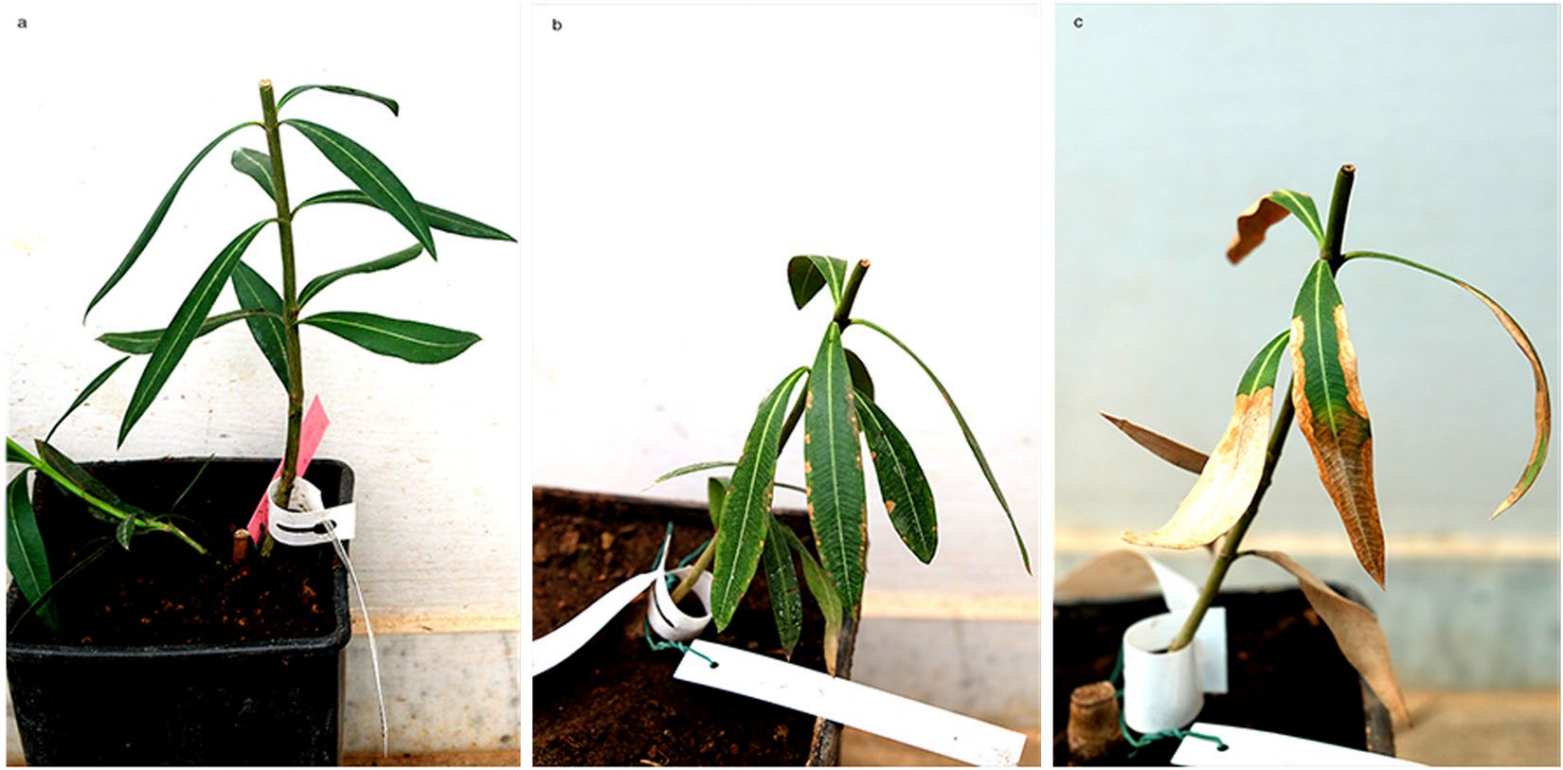
Inoculated oleander plants. Symptomless mock-inoculated oleander 14 months post inoculation; (**a**) leaf scorching in artificially inoculated oleander plants 10 months post inoculation (**b**) and 14 months post inoculation (**c**).

*X*. *fastidiosa* was readily detected at the IPs of all of the 10 inoculated plants of oleander 1 mpi, and was also present in 4 of 10 leaf petioles sampled at the node *ca*. 5–6 cm above the last IP. Eight of these plants were extensively colonized by the bacterium between 9 and 12 mpi (Experiment A). Typical marginal scorching of the leaves (Fig. 6) was observed in the inoculated plants as seen in naturally infected plants. Furthermore, the bacterium invaded the roots of all systemically infected plants tested at 12 mpi and was successfully re-isolated. Stunting and delayed flowering were observed in the course of the experiment on some infected oleanders (data not shown).

**Figure 7:**
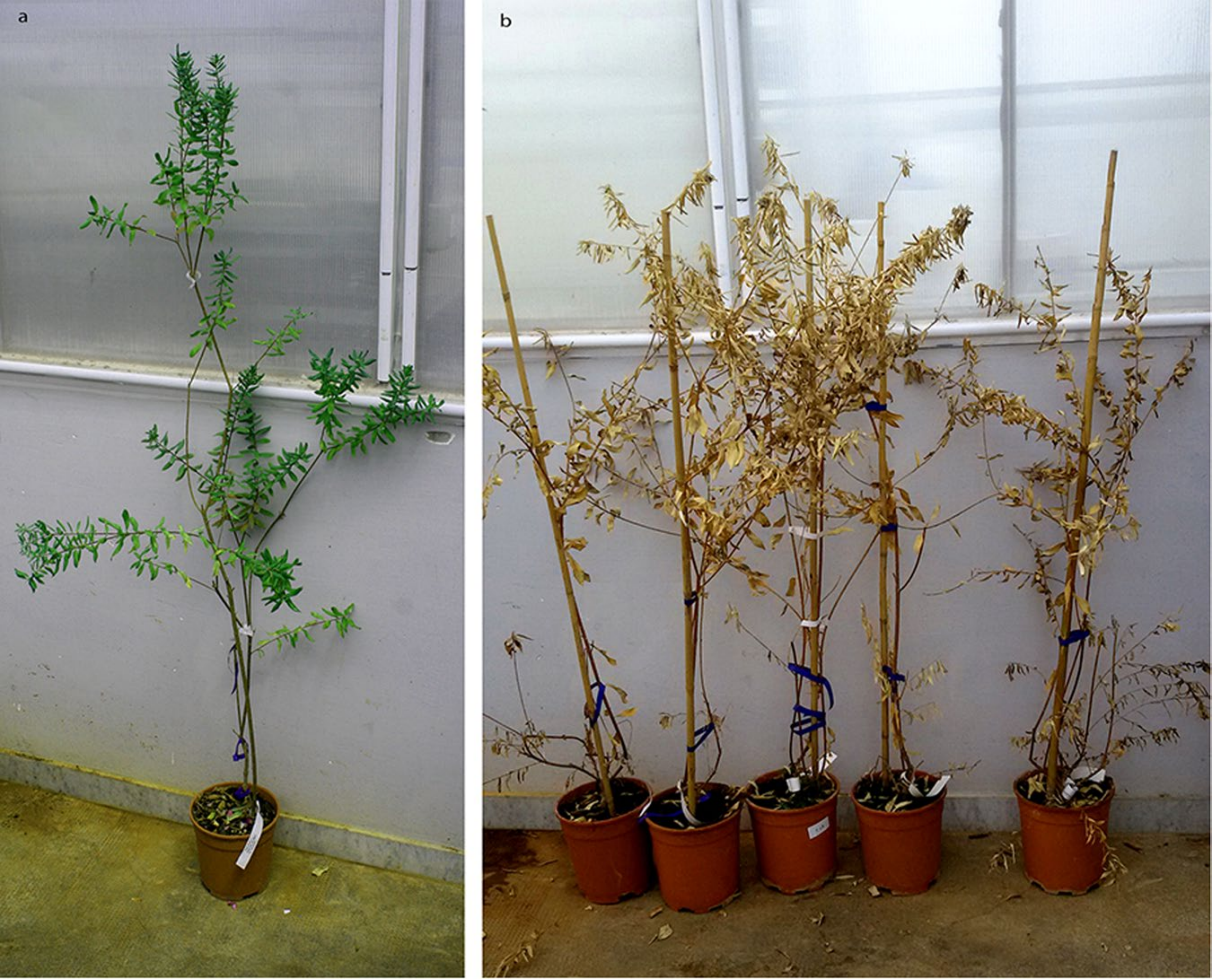
Extensive desiccation of artificially inoculated myrtle-leaf milkwort plants 20 months post inoculation. The mock-inoculated control is on the left end side.

Systemic infections and distinct symptoms were also obtained in all the artificially inoculated myrtle-leaf milkwort plants. Leaf scorching appeared 4–6 mpi in the medium-basal part of the canopy and involved the whole of it in 10 mpi. The bacterium invaded also the roots of all artificially infected plants which died within two years post inoculation (Fig. 7). Re-isolation of *X*. *fastidiosa* colonies from leaf petioles and stems was readily achieved on BCYE agar plates. Direct qPCR assays on scraped colonies produced consistent positive reactions for *X*. *fastidiosa*.

### Graft-transmission of *X*. *fastidiosa* to olives

Graft take was approximately 30% (15 grafted plants out of 48). Five of these plants originated from qPCR-negative cuttings and 10 from positive cuttings. Two and six months after grafting, consistent negative responses by qPCR came from the five plants derived from healthy cuttings (qPCR negative) whereas 6 and 10 positives, at 2 and 6 months after grafting, respectively, were from infected cuttings. Twelve months after grafting rootstocks, roots were also checked for bacterial presence, yielding results consistent with the presence of typical OQDS symptoms (Supplementary Fig. S2). None of these infected and symptomatic plants survived 16 months post-grafting, whereas plants from the qPCR-negative cuttings continued to grow and remained symptomless.

## Conclusion

*X*. *fastidiosa* is a xylem-restricted pathogen native to the Americas, where it is the causal agent of several detrimental diseases such as Pierce’s disease of grapevines^17^, citrus variegated chlorosis^18^, coffee leaf scorch^19^, blueberry leaf scorch^20,21^, pecan leaf scorch^22^, oleander leaf scorch^23^ and cherry leaf scorch^24^. Symptoms induced by *X*. *fastidiosa* may vary with the host species and the bacterial strain causing the infection. Broadly speaking, leaf-scorching is a most frequent alteration shown by a number of hosts such as shade trees, oleander, almond, cherry, pecan, coffee, blueberry, which may be followed by shoot and branch dieback. Leaf scorch is also the first symptom of Pierce’s disease in grapes, that appears on the leaves of the middle portion of the canes and progresses in both directions^25^. In citrus, *X*. *fastidiosa* induces variegated chlorosis, a disease characterized by interveinal chlorotic areas on the upper surface of the leaves and gummy lesions on the underside of the blade that turn brown and necrotic. Infected trees are stunted, and bear fruits of reduced size and quality^26^.

The finding of *X*. *fastidiosa* in the Old World was concomitant with the appearance of OQDS, a novel olive disease, first reported from southern Italy^27^, then from Argentina^5^ and Brazil^6^. The rapid expansion of OQDS in Apulia has caused substantial damage to the local olive and oil industry and the local economy, and raised major concerns for the neighbouring olive-growing Italian regions and the Mediterranean countries^12,28^.

Previous studies have demonstrated the susceptibility of olive, oleander and myrtle-leaf milkwort to the infection of the Salentinian strain of *X*. *fastidiosa* through vector-mediated transmission trials^10,29^. These experiments, while proving that *Philaenus spumarius* L. is a competent and the only ascertained vector of this strain, confirmed that these host plants are capable of supporting *Xylella* infections upon exposure to infective vector populations.

The isolation campaign, conducted in the numerous OQDS outbreaks emerged in the southern part of the Apulia region, resulted in the consistent detection and isolation of the bacterium from the symptomatic trees, further supporting the strong association between OQDS and *X*. *fastidiosa*.

Indeed, this is the first study that proves experimentally the relatedness of *X*. *fastidiosa* subsp. *pauca* strain De Donno with OQDS and diseases of oleander and myrtle-leaf milkwort, and demonstrates the ability of this bacterial strain to induce in these hosts the same symptoms observed in the field. In fact, mechanical and graft inoculations of *X*. *fastidiosa*-free olive plants induced wilting, dieback and death of these plants. The overall biological data support the strong causal relation between the bacterial infection and the presence of OQDS in infected olives, thus fulfilling Koch’s postulates.

The results of this work further extend the knowledge on the susceptibility of myrtle-leaf milkwort, a novel host species identified for the first time in the European territory. For this host, the Koch’s postulates were fulfilled upon mechanical inoculations of two strains of *X*. *fastidiosa* subspecies *multiplex* ^14^. For both strains, inoculated plants developed leaf scorching, whereas in our experiments with *X*. *fastidiosa* subsp. *pauca* strain De Donno, these symptoms were followed by extensive desiccations, dieback and death of the plants.

Infections in oleander, both under field or greenhouse conditions were similar to those previously described in Florida^30^.

As to host-pathogen interactions, relevant new information was gained:

(i) The latent period of the infection in inoculated young olive plants may exceed one year. Comparing inoculations of plants of different age, symptoms appeared earlier (8 mpi *vs* 12 mpi) in older (Experiment C) than in smaller and younger plants (Experiment A). Once a susceptible olive plant is systemically invaded by the bacterium (i.e. 9 mpi in the case of ‘Cellina di Nardò’) it takes at least 3–5 months for the symptoms to appear. Time course detection in inoculated plants, disclosed that colonization of olives is slower than in other hosts like grapevine^31^ and citrus^32^, which concurs with the long latent infection period in olive.
(ii) In agreement with field observations^33,34^, artificially inoculated olive cultivars responded differently as to timing of reaction and severity of symptoms. Thus, for instance, ‘Cellina di Nardò’ proved to be the most susceptible among the assayed cultivars (nine infected plants out of ten inoculated), showing the strongest symptomatic response and the highest bacterial population.
(iii) Systemic colonization of mechanically inoculated plants followed by active bacterial multiplication in the roots seem to have a bearing on symptom severity, hence on the differential response to infection. The highest rate of root colonization corresponded to the strongest symptoms expression in ‘Cellina di Nardò’, whereas the delayed colonization of ‘Leccino’ roots and the inconsistent presence of detectable bacterium in the roots of ‘Coratina’ and ‘Frantoio’ was associated with milder symptoms. This may be taken as an indication that *X*. *fastidiosa* encounters difficulties in colonizing the latter olive genotypes, with consequent erratic distribution in the canopy of the field-grown trees^34^.
(iv) Climatic conditions deeply impacted the outcome of *X*. *fastidiosa* infection. In our experiments the variable temperatures recorded in the net tunnels (Experiment B) appeared to be critical for successful host colonization and determining disease symptoms. Temperature effects were likely to be exacerbated by the use of small potted plants, that were more susceptible to temperature shifts than the adult olive trees in the field.

The overall result of this study, while proving the pathogenic role of *X*. *fastidiosa* subsp. *pauca* strain De Donno to olives and other susceptible hosts, clearly shows that the list of important plant diseases caused by *X*. *fastidiosa* continues to increase, and provides support to the need for implementing strategies, aimed at containing the spread of *X*. *fastidiosa* in the olive- growing areas. Finally, the outcome of the artificial inoculation of different olive cultivars calls for the promotion of research programs for the identification of resistant genetic traits in the olive germplasm.

## Materials and Methods

### Sampling olive trees for the presence of *X*. *fastidiosa*

Surveys were carried out in the Salento peninsula between 2014 and 2017 to identify olive groves with typical OQDS symptoms to be used for sampling. Whereas only a few OQDS foci were identified in summer 2014, more than 50 could be selected between May and January 2017 (Table 1, Supplementary Fig. S1), because of their rapid increase in number consequent to the uncontrolled spreading of the pathogen as officially declared by the Plant Health Authorities (http://eur-lex.europa.eu/legal-content/EN/TXT/PDF/?uri=CELEX:32016D0764&from=EN). The minimum distance between the closest foci was 2 km. In each of the 58 foci, one diseased grove was selected, and in each grove, from 3 to 4 ancient trees of ‘Ogliarola Salentina’ or ‘Cellina di Nardò’ were sampled by collecting from each 8–10 twigs next to symptomatic branches.

### Bacterial detection and isolation

DNA was extracted from leaf petioles following a standard CTAB-based procedure^35^, and the presence of bacterial DNA ascertained by quantitative real time PCR (qPCR)^36^. Cuttings 1 to 2-year-old and 0.4–0.8 cm in diameter were selected from one qPCR-positive tree representative of each selected grove and used for bacterial isolation in axenic culture. Thus, cuttings from 58 different trees were cut into pieces 8 to 10 cm long, washed under tap water, surface-sterilized in 2% sodium hypochlorite for 2 min, soaked in 70% ethanol for 2 min and rinsed three times in sterile water. Each piece was cut in half and squeezed at one end with a plier, while the other end was gently pressed on a buffered charcoal yeast extract (BCYE) growth medium^37^ to make 2–3 imprints. For each sample three BCYE plates were spotted (*ca*. 20–30 spots per plate), incubated for 3–4 weeks at 28 °C and periodically inspected for the growth of *Xylella* colonies. The recovered colonies were scraped and resuspended in potassium phosphate buffer, prior to being assessed by qPCR^36^ and re-plated in order to get individual colonies, which were then triple cloned and stored at −80 °C in 50% glycerol.

### Pathogenicity tests using *X*. *fastidiosa* colonies from axenic cultures

For pathogenicity tests one cultured bacterial isolate was selected following screening through multilocus sequence typing (MLST)^38^ the genetic profile of 20 isolates among those recovered from the most distant sampling sites^13^. All the isolates proved to belong to Sequence Type 53 (ST53). One of them, denoted “De Donno” (CFBP 8402), was retained as reference strain and used for mechanical inoculation tests on olives, oleander and myrtle-leaf milkwort. Its complete genome sequence, a DNA molecule 2,508,465 bp in size, has recently been determined^39^.

### Mechanical inoculation of *X*. *fastidiosa*

Inocula of isolate “De Donno” were prepared from 8- to 10-day-old colonies scraped from plates, dispersed in sterile potassium phosphate buffer (0.05 M, pH 7.2) as a turbid cell suspension of approximately 10^9^ cells/ml, and immediately used for needle inoculations^31^.

Three independent experiments (A, B, C) were carried out between October 2014 and May 2015. With Experiment A a set of 10 plants grown in a greenhouse at a temperature of 20–24 °C (winter) and 25–30 °C (summer) was inoculated in December, whereas with Experiment B the same set of plants was inoculated in October 2014, grown under a net tunnel in the OQDS-affected area. Plant species used for these two experiments included olives of four cultivars, oleander and myrtle-leaf milkwort. Olives of ‘Leccino’, ‘Frantoio’ and ‘Coratina’ were self-rooted plantlets 40–60 cm in size, whereas those of ‘Cellina di Nardò’ were grafted on olive seedlings.

Experiment C was specifically aimed at assessing the pathogenicity of the selected bacterial isolate to a set of 2-year-old, *ca*. 2 m tall grafted plants of ‘Cellina di Nardò’, the most OQDS-affected cultivar under field conditions. A total of 14 plants were inoculated on multiple shoots while 10 control plants were mock-inoculated. All plants were maintained under controlled conditions as with Experiment B.

For inoculation, a small drop of the bacterial suspension (10 µl) (PBS, for controls) was placed at the level of three consecutive nodes in the basal part of the stem, which was pricked 5–6 times with a sterile entomological needle. With myrtle-leaf milkwort and the 2-year-old ‘Cellina di Nardò’ 3–4 shoots per plant were inoculated, totaling 9–12 inoculation points (IP). Only one round of inoculations was performed for each set of plants.

### Detection of *X*. *fastidiosa* and symptom development in inoculated plants

All inoculated plants were periodically assayed for *X*. *fastidiosa* and symptom development was monitored over a period of almost two years. The appearance of wilting, shoot dieback and desiccations were constantly monitored and, for the olive plants, the intensity was determined by measuring the length of the shoot portions showing the alterations and compared to the overall length of the shoots present on each plant. In all plants, except for the myrtle-leaf milkwort, plant growth (total length of the shoots of each plant) at 12 and 24 mpi was assessed. For oleander, leaf scorching with marginal necrosis were recorded periodically.

#### Experiments A and B

The presence of *X*. *fastidiosa* was determined by qPCR 1, 3, 6, 9 and 12 mpi. Plants testing negative up to 12 mpi, were re-assayed in the following year. For the first sampling (1 mpi), one leaf petiole was collected from IPs and the node above the last IP for checking bacterial colonization. For subsequent samplings leaves were collected from different nodes above the IPs to follow the progression of bacterial colonization. However, at 12 mpi, the stem of three of the 10 inoculated individuals of each plant species/cultivar, was chopped into 12–15 cm segments for determining bacterial colonization. Isolation from each segment was also attempted, using the procedure previously described. At 12 and 24 mpi also roots were sampled and tested.

#### Experiment C

Samples were collected and qPCR assayed at 1 mpi, to determine bacterial multiplication at the site of inoculation, then at 7 mpi to assess bacterial movement in inoculated and non-inoculated shoots.

In all experiments, infection was considered systemic when *X*. *fastidiosa* was detected by qPCR in the petioles of the leaves collected at the 3^rd^−4^th^ node (*ca*. 7–10 cm) above the IP.

### Graft-transmission of *X*. *fastidiosa* to olives

Cuttings of a size suitable for grafting (6–7 mm in diameter) were collected from green branches of an OQDS-affected tree of ‘Ogliarola Salentina’, checked by isolation and qPCR for the presence of *X*. *fastidiosa*, and top-grafted on a total of 48 potted 3-year-old olive seedlings. After graft-take, the bacterial population was monitored in the new sprouts pushed by the scions, and in the rootstocks, to determine whether the bacterium had moved into them.

### Statistical analysis

Statistical analysis was performed with the CoStat version 6.204 (CoHort Software, CA, USA) and the SPSS 23.0 (IBM, Armonk, NY, USA) software programs. Comparisons among numeric data sets were conducted using the one-way or factorial repeated-measures ANOVA. Pairwise comparisons were made via Tukey’s HSD and Bonferroni’s post-hoc tests. Statistical significance was accepted for p-values < 0.05 α-level.

## Electronic supplementary material

supplementary figures

statistical analyses
