# Supplementary material for: Isolation and pathogenicity of Xylella fastidiosa associated to the olive quick decline syndrome in southern Italy
Source: Sci Rep. 2017 Dec 18;7:17723. doi: 10.1038/s41598-017-17957-z (PMC5735170; doi:10.1038/s41598-017-17957-z)
Supplement: Supplementary file 1 — supplementary figures [file 41598_2017_17957_MOESM1_ESM.doc]

**Isolation and pathogenicity of *Xylella fastidiosa* associated to the olive quick decline syndrome in southern Italy**

Saponari M^1 *^, Boscia D.^1^, Altamura G.^1^, Loconsole G.^2^, Zicca S. ^1^, D’Attoma G:^1,2^, Morelli M.^1^, Palmisano F.^3^, Saponari A. ^3^, Tavano D.^1^, Savino V.N.^2^, C. Dongiovanni^3^, Martelli G.P.^2^

^1^Consiglio Nazionale delle Ricerche-Istituto per la Protezione Sostenibile delle Piante (CNR-IPSP), Sede Secondaria di Bari, 70126 Bari, Italy

^2^Università degli Studi di Bari Aldo Moro, Dipartimento di Scienze della Pianta, del Suolo e degli Alimenti (DiSSPA), 70126 Bari, Italy

^3^Centro di Ricerca, Formazione e Sperimentazione in Agricoltura (CRSFA) “Basile Caramia”, 70010 Locorotondo (Bari), Italy

^*^Corresponding author: [maria.saponari@ipsp.cnr.it](mailto:maria.saponari@ipsp.cnr.it)


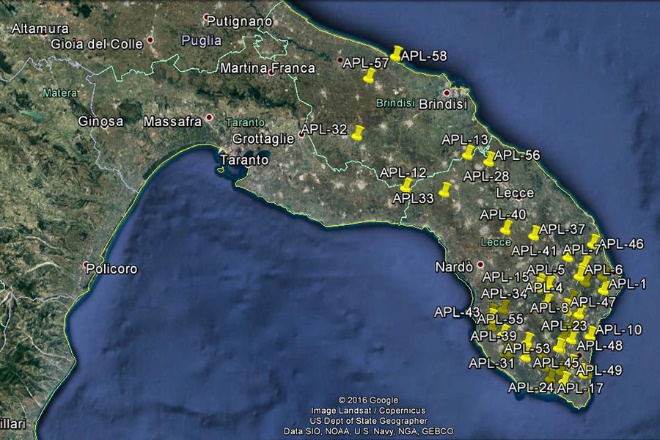


**Supplementary Figure S1**. Map of the Salentinian Peninsula (Apulia, southern Italy) with the sites in which olive trees affected by olive quick decline syndrome were identified and sampled for detection of *Xylella fastidiosa* between 2014 and early 2017. © 2016 Google, ImageLandsat/Copernicus, US Dept. of State Geographer.


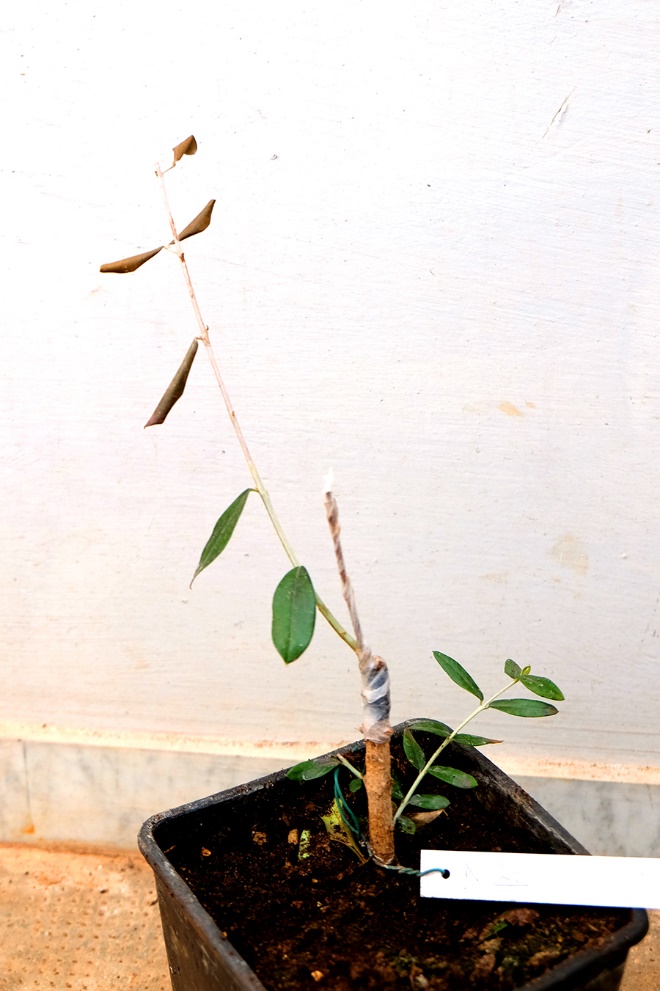


**Supplementary Figure S2.** Grafted olive plant obtained from a *Xylella*-infected scion. After graft take, the new developed shoot started to show typical desiccation (approximately 10 month after graft uptake).
