# Supplementary material for: Isolation and pathogenicity of Xylella fastidiosa associated to the olive quick decline syndrome in southern Italy
Source: Sci Rep. 2017 Dec 18;7:17723. doi: 10.1038/s41598-017-17957-z (PMC5735170; doi:10.1038/s41598-017-17957-z)
Supplement: Supplementary file 2 — statistical analyses [file 41598_2017_17957_MOESM2_ESM.doc]

**Isolation and pathogenicity of *Xylella fastidiosa* associated to the olive quick decline syndrome in southern Italy**

Saponari M^1 *^, Boscia D.^1^, Altamura G.^1^, Loconsole G.^2^, Zicca S. ^1^, D’Attoma G:^1,2^, Morelli M.^1^, Palmisano F.^3^, Saponari A. ^3^, Tavano D.^1^, Savino V.N.^2^, C. Dongiovanni^3^, Martelli G.P.^2^

^1^Consiglio Nazionale delle Ricerche-Istituto per la Protezione Sostenibile delle Piante (CNR-IPSP), Sede Secondaria di Bari, 70126 Bari, Italy

^2^Università degli Studi di Bari Aldo Moro, Dipartimento di Scienze della Pianta, del Suolo e degli Alimenti (DiSSPA), 70126 Bari, Italy

^3^Centro di Ricerca, Formazione e Sperimentazione in Agricoltura (CRSFA) “Basile Caramia”, 70010 Locorotondo (Bari), Italy

^*^Corresponding author: [maria.saponari@ipsp.cnr.it](mailto:maria.saponari@ipsp.cnr.it)

**Supplementary Table S1**

Factorial repeated-measures ANOVA comparing the effect of cultivar (Cellina di Nardò, Coratina, Frantoio and Leccino) between factor and time post-inoculation (1 mpi, 3 mpi, 6 mpi, 9 mpi, 12 mpi, 24 mpi) within factor, as well as of their interaction, on the distance of *Xylella fastidiosa* detection in plants used in Experiment A. Post hoc pairwise comparisons were performed according to the Bonferroni procedure. The significance level α was set at 5 % for F- and p-values. A p-value lower than 0.05 (in bold) indicates significant differences..

| **Source** | **df** | **F** | **p-value** | **Partial ƞ^2^** | |
| --- | --- | --- | --- | --- | --- |
| ***within-subjects effects*** | | | | | |
| Time | 5 | 69.786 | **<0.0005** | 0.660 | |
| Time * Cultivar | 15 | 1.940 | **0.022** | 0.139 | |
| Error | 180 |  |  |  | |
| ***between-subjects effects*** | | | | | |
| Cultivar | 3 | 3.409 | **0.028** | 0.221 | |
| Error | 36 |  |  |  | |
| ***Bonferroni post-hoc pairwise comparisons*** | | | | | |
| **Time** | **Cultivar (I)** | **Cultivar (J)** | **Mean Difference (I-J)** | **Std. Error** | **p-value** |
| 1 mpi | Cellina di Nardò | Coratina | 0.500 | 0.261 | 0.383 |
|  |  | Frantoio | 0.400 | 0.261 | 0.808 |
|  |  | Leccino | 0.400 | 0.261 | 0.808 |
| 3 mpi | Cellina di Nardò | Coratina | 1.300 | 0.486 | 0.067 |
|  |  | Frantoio | 1.000 | 0.486 | 0.283 |
|  |  | Leccino | 0.700 | 0.486 | 0.953 |
| 6 mpi | Cellina di Nardò | Coratina | 4.000 | 1.117 | **0.006** |
|  |  | Frantoio | 3.400 | 1.117 | **0.026** |
|  |  | Leccino | 3.200 | 1.117 | **0.041** |
| 9 mpi | Cellina di Nardò | Coratina | 4.700 | 1.348 | **0.008** |
|  |  | Frantoio | 3.900 | 1.348 | **0.039** |
|  |  | Leccino | 3.700 | 1.348 | 0.056 |
| 12 mpi | Cellina di Nardò | Coratina | 5.000 | 1.972 | 0.094 |
|  |  | Frantoio | 2.000 | 1.972 | 1.000 |
|  |  | Leccino | 1.000 | 1.972 | 1.000 |
| 24 mpi | Cellina di Nardò | Coratina | 4.000 | 1.986 | 0.309 |
|  |  | Frantoio | 2.000 | 1.986 | 1.000 |
|  |  | Leccino | 1.000 | 1.986 | 1.000 |

**Supplementary Table S2**

ANOVA one-way comparing the average bacterial concentration among the four cultivars (Cellina di Nardò, Coratina, Frantoio and Leccino), as measured in plants reported in Table 4. Post hoc pairwise comparisons were performed using the Tukey HSD test. The significance level α was set at 5 % for F- and p-values. A p-value lower than 0.05 (in bold) indicates significant differences. A separate analysis was conducted for each type of tissue analysed (i.e. stem or leaf petioles).

**Stem**

| **Source** | **df** | | **F** | | | **p-value** | | **Partial ƞ^2^** | | | |
| --- | --- | --- | --- | --- | --- | --- | --- | --- | --- | --- | --- |
| ***between-subjects effects*** | | | | | | | | | | | |
| Cultivar | | 3 | | 7.146 | | | **0.005** | | | 0.641 | |
| Error | | 12 | |  | | |  | | |  | |
| ***Tukey HSD post-hoc pairwise comparisons*** | | | | | | | | | | | |
| **Cultivar (I)** | | **Cultivar (J)** | | | **Mean Difference (I-J)** | | | | **Std. Error** | | **p-value** |
| Cellina di Nardò | | Leccino | | | 6.688E+06 | | | | 1.693E+06 | | **0.009** |
|  |  | Frantoio | | | 6.281E+06 | | | | 1.693E+06 | | **0.014** |
|  |  | Coratina | | | 6.187E+06 | | | | 1.693E+06 | | **0.015** |
| Leccino | | Frantoio | | | -4.077E+05 | | | | 1.693E+06 | | 0.995 |
| Leccino | | Coratina | | | -5.008E+05 | | | | 1.693E+06 | | 0.991 |
| Coratina | | Frantoio | | | 9.312E+04 | | | | 1.693E+06 | | 1.000 |

**Leaf petioles**

| **Source** | **df** | | **F** | | **p-value** | **Partial ƞ^2^** | | |
| --- | --- | --- | --- | --- | --- | --- | --- | --- |
| ***between-subjects effects*** | | | | | | | | |
| Cultivar | 3 | | 22.444 | | **<0.005** | 0.849 | | |
| Error | 12 | |  | |  |  | | |
| ***Tukey HSD post-hoc pairwise comparisons*** | | | | | | | | |
| **Cultivar (I)** | | **Cultivar (J)** | | **Mean Difference (I-J)** | | | **Std. Error** | **p-value** |
| Cellina di Nardò | | Leccino | | 3.241E+06 | | | 4.974E+05 | **<0.0005** |
|  |  | Frantoio | | 3.327E+06 | | | 4.974E+05 | **<0.0005** |
|  |  | Coratina | | 3.419E+06 | | | 4.974E+05 | **<0.0005** |
| Leccino | | Frantoio | | 8.668E+04 | | | 4.974E+05 | 0.865 |
| Leccino | | Coratina | | 1.781E+05 | | | 4.974E+05 | 0.727 |
| Coratina | | Frantoio | | -9.137E+04 | | | 4.974E+05 | 0.857 |

**Supplementary Table S3**

Results of pairwise comparisons using a Mann-Whitney test between average bacterial concentration detected in ‘Cellina di Nardò’ plants grown under a net tunnel (Experiment B) and those grown in a greenhouse (Experiment A). The significance level α was set at 5 % for F- and p-values. A p-value lower than 0.05 (in bold) indicates significant differences. The hypothesis of a normal distribution was preliminarily rejected according to the results of Kolmogorov-Smirnov and Shapiro-Wilk tests, here reported.

**Test of Normality**

| **Test** | **Statistic** | **df** | **p-value** |
| --- | --- | --- | --- |
| Kolmogorov-Smirnov | 0.289 | 29 | <0.0005 |
| Shapiro-Wilk | 0.552 | 29 | <0.0005 |

**Mann-Whitney Test**

| **U-value** | **Z** | **p-value (1-tailed)** |
| --- | --- | --- |
| 7.000 | -3.338 | **0.0005** |

**Supplementary Table S4**

ANOVA one-way comparing the average percentage of canopy affected by desiccation among the four cultivars (Cellina di Nardò, Coratina, Frantoio and Leccino), as measured in plants reported in Table 5. Post hoc pairwise comparisons were performed using the Tukey HSD test. A p-value lower than 0.05 (in bold) indicates significant differences. The significance level was set at 5 % (α = 0.05) for F- and p-values. A separate analysis was conducted for each time point post-inoculation (i.e. 12 or 24 mpi).

**12 mpi**

| **Source** | **df** | **F** | **p-value** | **Partial ƞ^2^** |
| --- | --- | --- | --- | --- |
| ***between-subjects effects*** | | | | |
| Cultivar | 3 | 1.914 | 0.198 | 0.389 |
| Error | 9 |  |  |  |

**24 mpi**

| **Source** | **df** | | **F** | | **p-value** | **Partial ƞ^2^** | | |
| --- | --- | --- | --- | --- | --- | --- | --- | --- |
| ***between-subjects effects*** | | | | | | | | |
| Cultivar | 3 | | 12.872 | | **<0.0005** | 0.617 | | |
| Error | 24 | |  | |  |  | | |
| ***Tukey HSD post-hoc pairwise comparisons*** | | | | | | | | |
| **Cultivar (I)** | | **Cultivar (J)** | | **Mean Difference (I-J)** | | | **Std. Error** | **p-value** |
| Cellina di Nardò | | Coratina | | 78.346 | | | 13.339 | **<0.0005** |
|  |  | Frantoio | | 62.369 | | | 13.339 | **0.001** |
|  |  | Leccino | | 43.680 | | | 13.339 | **0.016** |
| Leccino | | Frantoio | | 18.689 | | | 13.339 | 0.511 |
| Leccino | | Coratina | | 34.665 | | | 13.339 | 0.070 |
| Coratina | | Frantoio | | -15.977 | | | 13.339 | 0.634 |
